# Identification and characterization of the karrikins signaling gene *SsSMAX1* in *Sapium sebiferum*

**DOI:** 10.7717/peerj.16610

**Published:** 2023-12-08

**Authors:** Fang Ni, Faheem Afzal Shah, Jie Ren

**Affiliations:** 1 Anhui Wenda University of Information Engineering, Hefei, Anhui, China; 2 Institute of Agricultural Engineering, Anhui Academy of Agricultural Sciences, Hefei, Anhui, China

**Keywords:** SMAX1, Seed dormancy, Seedlings development, Hypocotyl length, Root length

## Abstract

*SUPPRESSOR OF MAX2 LIKE 1 (SMAX1)* is a member of the *SUPPRESSOR of MAX2 1‑LIKE* family of genes and is known as a target protein of *KARRIKIN INSENSITIVE2 (KAI2)-MORE AXILLARY BRANCHES2 (MAX2)*, which mediates karrikin signaling in *Arabidopsis*. *SMAX1* plays a significant role in seed germination, hypocotyl elongation, and root hair development in *Arabidopsis*. SMAX1 has not yet been identified and characterized in woody plants. This study identified and characterized *SsSMAX1* in *Sapium sebiferum* and found that *SsSMAX1* was highly expressed in the seed, hypocotyl, and root tips of *S. sebiferum*. *SsSMAX1* was functionally characterized by ectopic expression in *Arabidopsis*. *SsSMAX1* overexpression lines of *Arabidopsis* showed significantly delayed seed germination and produced seedlings with longer hypocotyl and roots than wild-type and *Atsmax1* functional mutants. *SsSMAX1* overexpression lines of *Arabidopsis* also had broader and longer leaves and petioles than wild-type and *Atsmax1*, suggesting that *SsSMAX1* is functionally conserved. This study characterizes the *SMAX1* gene in a woody and commercially valuable bioenergy plant, *Sapium sebiferum*. The results of this study are beneficial to future research on the molecular biology of woody plants.

## Introduction

Karrikins (KARs) are butenolides, which are formed by the burning of biomass. Karrikins enhance seed germination (Nelson et al., 2009; Flematti et al., 2004; Nelson et al., 2012), promote seedling response to light in the modal *Arabidopsis thaliana*, and improve seedling vigour in many plants (Jain, Kulkarni & van Staden, 2006; Kulkarni et al., 2006; Nelson et al., 2010; van Staden et al., 2006). In plants, the putative receptors for KARs and Strigolactones (SLs) are the paralogues a/b-hydrolases KARRIKIN INSENSITIVE2 (KAI2) and DWARF14 (D14), respectively. These paralogues are ancient and exist in all angiosperms (Waters et al., 2012, 2013). *KAI2* and *D14* require common catalytic triad residues (Ser95-His246-Asp217) for binding to KARs and SLs, respectively (Hamiaux et al., 2012; Hidemitsu et al., 2013; Li et al., 2013; Megumi et al., 2013; Rohan et al., 2013; Toh et al., 2015; Xia et al., 2013). SL and KAR signaling are both dependent on the activity of *MAX2*, an F-box containing protein that forms an Skp-Cullin-F-box (SCF) complex (Nelson et al., 2011; Stirnberg, Furner & Ottoline Leyser, 2007; Stirnberg, van De Sande & Leyser, 2002). The SCF complexes have ubiquitin moieties that bind to the target proteins, degrading the target proteins by the 26S proteasome (Somers & Fujiwara, 2009). Ligand binding or hydrolysis in *MAX2* might encourage conformational receptor variations, which may change *MAX2’s* interactions with downstream signaling protein partners (Hamiaux et al., 2012). The functional mutation of *Atmax2* showed various phenotypes, such as delayed seed germination, elongated hypocotyl, narrow leaves, and more axillary branches in *Arabidopsis*. The phenotype differences between strigolactones insensitive *D14* (more axillary branches) and *KARRIKINS INSENSITIVE* 2 (*KAI2)* phenotypes (seed germination, hypocotyl length, and leaf width) indicate that SLs and KARs control separate aspects of *MAX2*-dependent functions (Waters et al., 2012). These studies all suggest that the karrikin and strigolactone signaling pathways are *MAX2*-dependent but regulate different aspects of plant life.

The karrikins signaling pathway requires the deactivation of repressor proteins following hormone perception by ubiquitylation and subsequent proteasomal degradation (Nelson et al., 2012, 2010, 2011; Soundappan et al., 2015; Stanga, Morffy & Nelson, 2016; Stanga et al., 2013; Waters et al., 2012). Forward genetic screening has identified KARs signaling repressors (Soundappan et al., 2015; Stanga, Morffy & Nelson, 2016; Stanga et al., 2013; Wang et al., 2015; Zhou et al., 2013), which are members of the same SUPPRESSOR OF MAX2-like (SMXL) family. The SMXL family includes three clades in seed plants: (1) *SMAX1* and *SMXL2* for KARs signaling, (2) *SMAX* 6, *SMAX* 7, and *SMAX* 8 for strigolactones signaling, and (3) *SMAX*3, *SMAX*4, and *SMAX*5 acting in an unidentified signaling module that regulates phloem formation but is independent of strigolactones and KARs signaling (Soundappan et al., 2015; Stanga, Morffy & Nelson, 2016; Stanga et al., 2013; Walker et al., 2019; Wallner et al., 2017; Wang et al., 2015). The strigolactones signaling repressors, such as SMXL6, SMXL7, and SMXL8, and D53 proteins are ubiquitylated and degraded by proteasomes upon ligand sensing (Jiang et al., 2013; Soundappan et al., 2015; Walker et al., 2019; Zhou et al., 2013). It has also recently been discovered that SMAX1 and SMXL2 proteins are degraded by the interaction of KAI2 and MAX2 proteins (Khosla et al., 2020; Wang et al., 2020). The role of *SMAX1* and *SMXL2* in plant development has been genetically studied in *Arabidopsis*, where *smax1smxl2* double mutants display mild, *kai2*-opposing phenotypes, such as slightly faster seed germination, shorter hypocotyls, bigger cotyledons, and longer root hairs (Stanga, Morffy & Nelson, 2016; Villaécija-Aguilar et al., 2019). The *SUPPRESSER OF MAX2 LIKE 1 (SMXL1)* protein is the third class of putative target genes that have been identified. Functional mutant screening for SUPPRESSOR OF *MAX2-like* proteins in *Arabidopsis* led to the discovery of *SMAX1*, which regulates seed germination, seedling growth, leaf shape, and size (Stanga et al., 2013). A recent study provided a detailed description of SMXL family genes in a model woody plant, *P. trichocarpa*, but only characterized *PtSMXL7*, a signaling gene of strigolactones (Sun et al., 2023). The characterization of *SMAX1* in woody plants has not yet been investigated.

Chinese tallow (*Sapium sebiferum* L.), which belongs to the Euphorbiaceae family, is native to eastern Asia (Esser, 2002). Its fruits produce a highly-saturated fatty acid in the tallow layer and highly-unsaturated oil in the seed (Boldor et al., 2010). Tallow is used for manufacturing candles, soap, cloth, and fuel, and the seed oil can be used for making varnishes and paints (Brooks et al., 1987; Jeffrey & Padley, 1991). A single mature *S. sebiferum* tree produces many seeds, and is estimated to produce 4,700 liters of oil per hectare, annually. The average commercial yields of *S. sebiferum* far exceed traditional oilseed crops (Boldor et al., 2010; Webster, Jenkins & Jose, 2006). *S. sebiferum* is also popular because of its colorful autumn foliage, and has become a popular species for landscaping and as a source of biodiesel (Gao et al., 2016).

*S. sebiferum* seeds are dormant and require long stratification times to start normal germination. Seed germination, which is essential to the plant life cycle because it determines plant survival and reproductive success, is regulated by different hormones and signaling compounds, such as ABA, GA, ethylene, and karrikins (Carbonnel et al., 2020; Dekkers et al., 2016; Lee et al., 2002; Nelson et al., 2012). Seed culturing is an easy and widely-used method of commercial propagation of many plant species, including many bio-energy plants, so an efficient seed germination assay is needed by both researchers and nursery growers. *S. sebiferum* is best propagated through the seed, but *S. sebiferum’*s poor seed germination rate due to deep dormancy has limited the use of this species (Conway, Smith & Bergan, 2000). Only a few studies are available to understand and promote *S. sebiferum* seed germination. One study found that *S. sebiferum* seeds exposed to cold water for 72 h showed 10% seed germination (Li et al., 2012). Another study found that the dormancy of tallow tree seeds could be overcome by soaking the seeds in GA3, followed by 100 days of cold stratification (Li et al., 2012). A separate study explored the effect of KAR_1_ on *Sapium sebiferum* seed germination (Shah et al., 2020). In the karrikins pathway, *SMAX1* has been found to be abundant in seeds and to play a significant role in seed germination and seedling development. Despite this discovery, it is still unknown whether *SMAX1* functions are conserved in a perennial woody plant like *S. sebiferum*. This study analyzed the ectopic expression of *SsSMAX1* in *Arabidopsis* to confirm whether the functions of *SsSMAX1* are conserved. *SSMAX1* was identified by blasting *AtSMAX1* into a local blast library created by an already available *S. sebiferum* transcriptome. Ectopic expression of *SSMAX1* in *Arabidopsis* was phenotypically compared with loss-of-function mutants of *SMAX1* and wild-type *Arabidopsis*.

## Materials and Methods

### Bioinformatic analysis

The 3D structure of the SsMAX1 protein was predicted by the Phyre2 web portal for protein modeling, prediction, and analysis (http://www.sbg.bio.ic.ac.uk/phyre2; Kelley et al., 2015). The *Arabidopsis thaliana SMAX1* (AT5G57710) gene sequence was downloaded from TAIR (https://www.arabidopsis.org). *Arabidopsis thaliana, Populus trichocarpa* (XP_002324496.2), and *Sapium sebiferum SMAX1* gene sequences were aligned using the residue substitution matrix in the *AlignX* in Vector NTI Advance 11.0. For phylogenetic tree construction, *Arabidopsis* (AT5G57710), *P. trichocarpa* (XP_002324496.2), *Nicotiana tobacum* SMAX1 (XP_016511300.1), *Carica papaya SMAX1* (XP_021895142.1), *Oryza sativa* D53 (NP_001410055.1), and *Sapium sebiferum* SMAX1 protein sequences were aligned using the default settings of the *ClustalW* in MEGA11 software. After the alignment of *proteins*, the alignment results were saved in MEGA format, and a phylogenetic tree was developed by a neighborhood joining method using the Poisson model with 500 bootstrap replications in MEGA11 software.

### Cloning of *Sapium sebiferum SMAX1* gene

A local blast library was built in NCBI blast-2.2.31 using a fasta file of *S. sebiferum* flower-bud transcriptome (accession: SRX656554; Yang et al., 2015). After constructing the local blast library, the complete sequence of the *SsSMAX1* gene was obtained using the *Arabidopsis* SMAX1 amino-acids sequence as an input query in the tblastn function of NCBI blast-2.2.31 (Camacho & Madden, 2013). The *Arabidopsis SMAX1* (AT5G57710) amino-acids sequence was obtained from TAIR (https://www.arabidopsis.org/). The full-length sequence of *SsSMAX1* with the translated amino acid sequence is given in Table S1. The full-length open reading frame (ORF) of the *SsSMAX1* gene was found using the NCBI ORF finding tool. Gene-specific primers were designed by Primer Premier 5 to amplify the full-length ORF of *SsSMAX1*. The T_m_ of the primers was between 62.0 and 65.0 ° C; a list of all primers is given in Table S1. The gene cloning method is fully described in Fig. S1.

### Expression vector design

After cloning the full-length sequence of *SsSMAX1*, the PCR product was aligned with a *pEASY®*-Blunt Cloning Vector (TransGen Biotech Co., Beijing, China). The ORF of the *SsSMAX1* gene was sequenced and confirmed by Sangon Biotech (Shanghai) Co., Ltd. The cloned gene sequence in a *pEASY®*-Blunt Cloning Vector was double digested at Sal1 from the start, and Sma1 from the stop codon site, and then inserted into the expression vector, pOCA30, which was also double-digested at the same restriction sites. A map of the expression vector is drawn in Fig. S1. The expression vector was transformed to the Agrobacteria EHA105 strain. The floral dip method was used for gene transformation in *Arabidopsis* (Zhang et al., 2006). Seeds of the T4 homozygous *SsSMAX1* line (two lines) were selected for further experiments.

### Plant materials and growth conditions

*Arabidopsis* wild-type Columbia-0 (Col-0) and mutant *Atsmax1* were obtained from the *Arabidopsis* Biological Resources Center (Columbus, Ohio). Seeds of the T4 homozygous *SsSMAX1* lines (two lines) were selected for further experiments. Seeds of wild-type plants, *Atsmax1* and *SsSMAX1*, were surface sterilized by serial washing with 70% (v/v) ethanol for 2 min, then with 10% (v/v) NaClO for 10 min, and then washed three times with double distilled water. The sterilized seeds were plated on ½ Murashige and Skoog (MS) medium supplemented with 1% (w/v) sucrose plus 0.8% (w/v) agar and placed at 4 °C for 2 days. Seeds were germinated in a 16 h/8 h photoperiod at 22 °C, approximately 150 mmol photons m^2^/s^2^. Seven-day-old *Arabidopsis* seedlings were transferred from ½ MS medium to soil and grown in a growth room at 22 °C, approximately 150 mmol photons m^2^/s^2^ with 16 h/8 h (long-day conditions) photoperiods.

### Seed germination, hypocotyl length, and growth parameters analysis

The seed germination analysis was performed in 10 × 10 Petri plates. Each experiment was repeated five times, with 30 seeds of each genotype. Germinated seeds were counted starting 24 h after seeds were placed in a growth room. For hypocotyl and root length measurement, seeds were sown in 10 × 10 plates with grids. Pictures were taken after seven days of seed germination; ImageJ software measured root and hypocotyl length (15 seedlings). Leaf length (15 leaves per plant) was measured using ImagJ by taking leaf pictures of three different 45-day-old plants.

### Primer design, RNA extraction, cDNA synthesis, and RT–qPCR conditions

*SsSMAX1* gene’s full-length CDS and protein sequences are available in Document S1. Primers used for qPCR were designed with Primer Premier 6. The T_m_ of the primers was between 59.0 °C and 61.0 °C; a list of all primers is given in Table S1. For the gene expression analysis in the different tissues of *S. sebiferum*, samples (three biological replicates) were taken from the *S. sebiferum* tree growing at Anhui Academy of Agricultural Sciences. To measure SsSMAX1 expression in overexpression lines, leaf samples (three biological replicates) were taken from 15-day-old seedlings of WT, OE1, and OE2 *Arabidopsis*. Samples were frozen in liquid nitrogen and stored at −80 °C. RNA was extracted using an E.Z.N.A® plant RNA extraction kit (OMEGA Pro -TEK) based on the manufacturer’s instructions. Five hundred ng RNA of each sample was reverse transcribed using cDNA synthesis SuperMix (TransGen Biotech, Beijing, China) based on the manufacturer’s instructions. The cDNA samples were diluted 25X with sterile water. For each 20 microliter (μL) reaction of qPCR, 9 μL of cDNA, 10 μL of the 2X QuantiNova SYBR Green PCR Master Mix (QIAGEN, Hilden, Germany), and 0.5 μL of each primer were added to a final total volume of 20 μL. The qRT–PCRs were run on a Light Cycler®96 (Roche, Basel, Switzerland). The qPCR program consisted of two steps: the first step at 95.0 ° C for 3 min, and the second step had 45 cycles alternating between 15 s at 95.0 °C, 15 s at 60.0 °C, and 15 s at 72.0 °C.

### Statistical analysis

Excel 2013 was used to arrange the data and R Studio 1.1.383 was used for the statistical analyses. The data were represented as mean ± standard deviation. Results from the different treatments were analyzed separately. The wild-type *Arabidopsis* was used as the control group for each set of experiments. The results of overexpression lines (OE1 and OE2) and *Atsmax1* were compared with the results of wild-type *Arabidopsis*. The significance of treatments was tested by one-way analysis of variance (ANOVA). Tukey tests were used to identify significant differences between pairs of means at *p* < 0.05.

## Results

### Bio-informatics analysis of SsSMAX1

**Figure 1:**
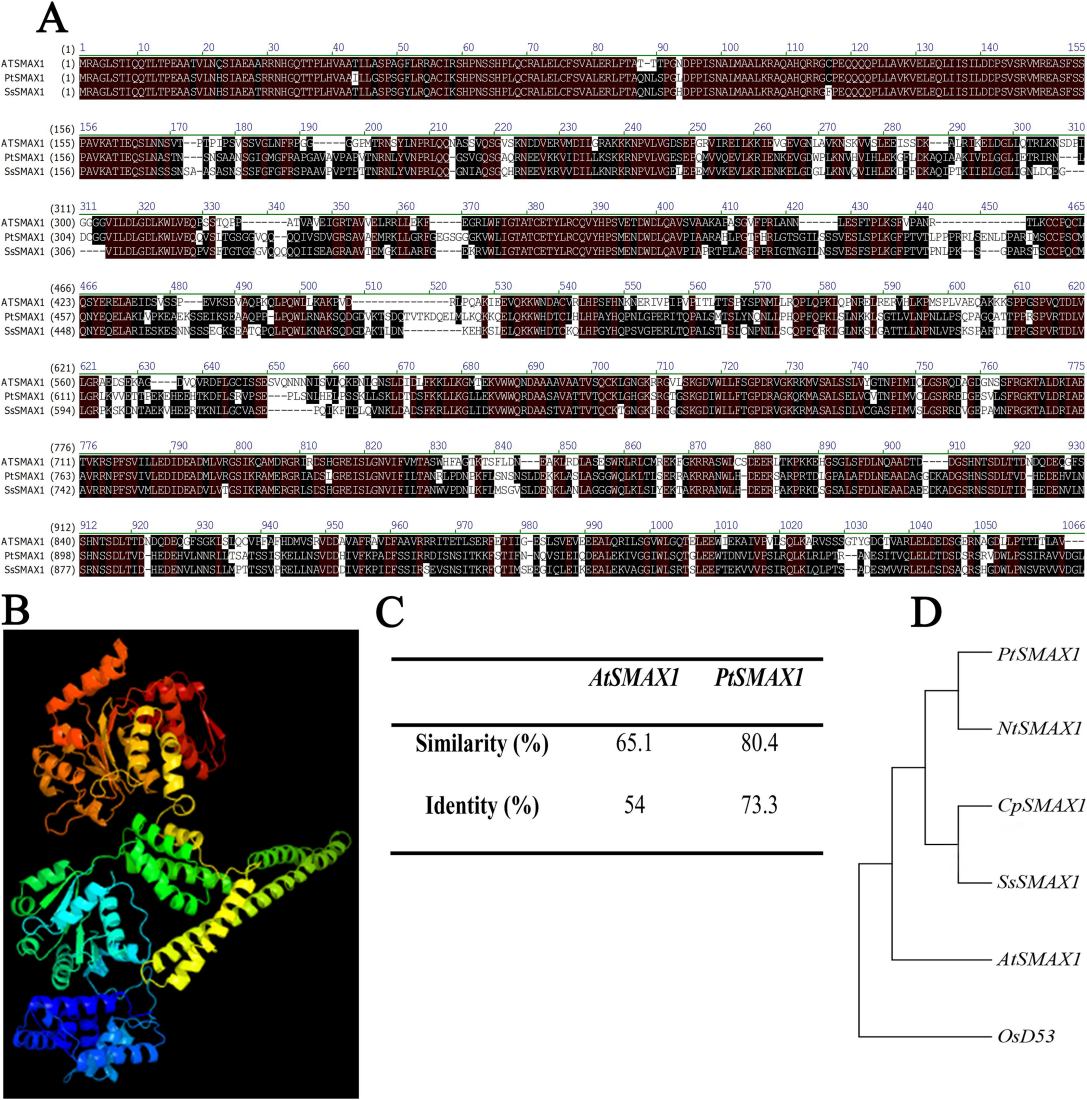
Bio-informatics analysis of *SsSMAX1*. (A) *Sapium sebiferum SMAX1* protein alignment with *Arabidopsis* and *Populus SMAX1* protein. (B) Similarity and identity of *SsSMAX1* with *AtSMAX1* and *PtSMAX1*. (C) The ribbon diagram of the tertiary (three-dimensional) structure of SsSMAX1. (D) Phylogenetic tree of *SsSMAX1* protein.

SsSMAX1 showed significant alignment with the SMAX1 proteins of modal plants like *Arabidopsis thaliana* and *Populus trichocarpa* (Fig. 1A**)**. The three-dimensional structure showed that SsSMAX1 contained the loops and turns, alpha helix, and beta sheets (Fig. 1B). Protein alignments calculated by residue substitution matrix showed that *SsSMAX1* is 65.1% similar to *AtSMAX1* and 80.4% identical to *PtSMAX1* (Fig. 1C). The phylogenetic tree showed that *SsSMAX1* has the same clad as papaya *CpSMAX1;* these proteins lay subclade to *Nicotiana tobacum SMAX1. SsSMAX1* was a neighbour to *AtSMAX1* (Fig. 1D).

**Figure 2:**
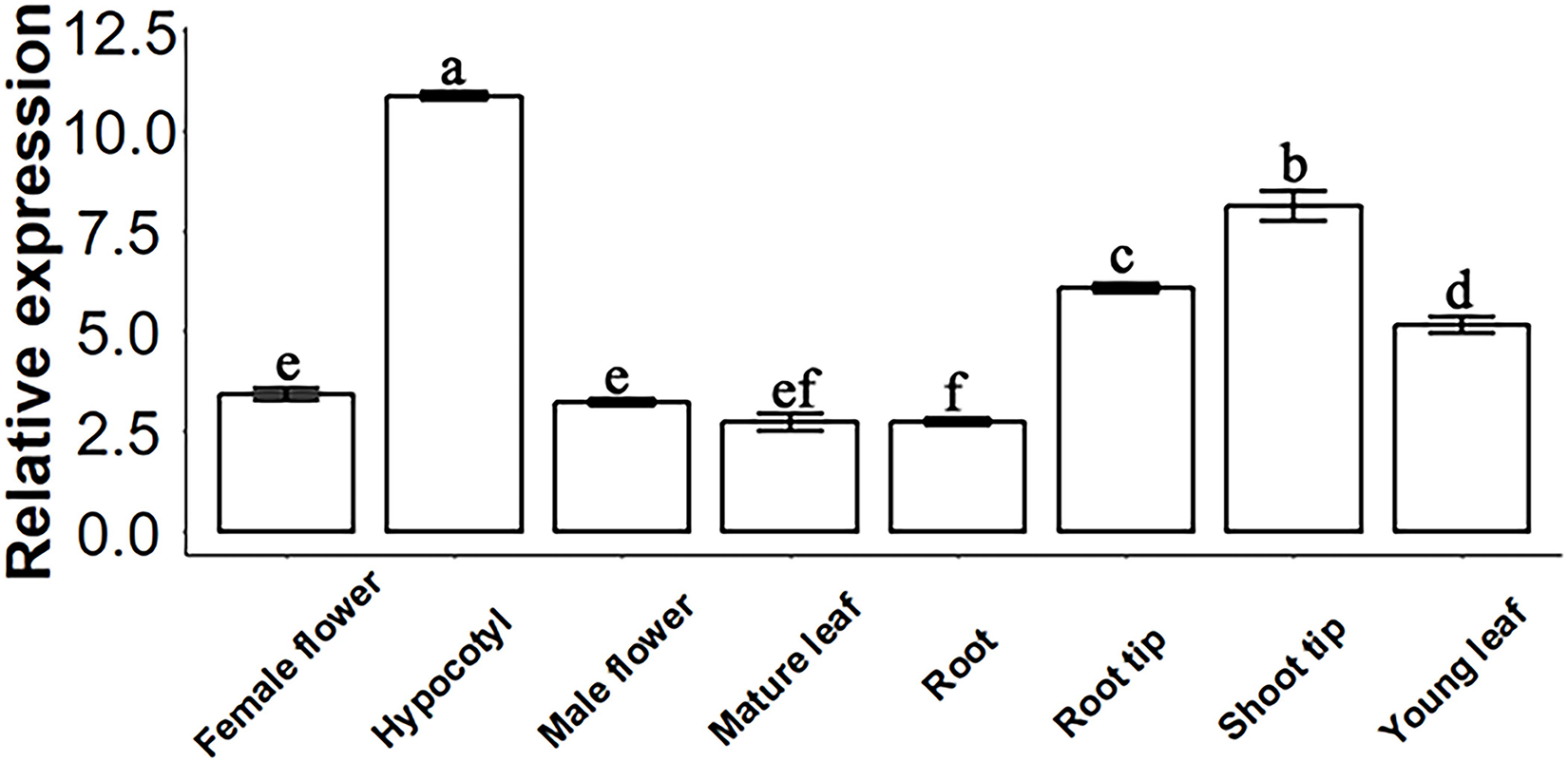
*SsSMAX1* relative expression in different organs and tissue in *Sapium sebiferum*. Gene expression determination by qPCR. Data represent the 2^−∆∆Ct^ value of each gene. *SsACTIN2* was used as a reference gene. Data were statistically analysed by one-way ANOVA, and multiple comparisons were made with HSD Tuckey’s test at *p* = 0.5 significant level (*n* = 3).

### SsSMAX1 distribution in *Sapium sebiferum*

The molecular function of a gene can be predicted by its expression levels in different tissues of *Sapium sebiferum*. The results of an analysis of expression levels showed that *SsSMAX1* was highly expressed in hypocotyl, root tip, shoot tip, and young leaf in *Sapium sebiferum* (Fig. 2), indicating that *SsSMAX1* is involved in the regulation of leaf shape, hypocotyl development, and root architecture development.

### SsSMAX1 involvement in seed germination and seedling development

**Figure 3:**
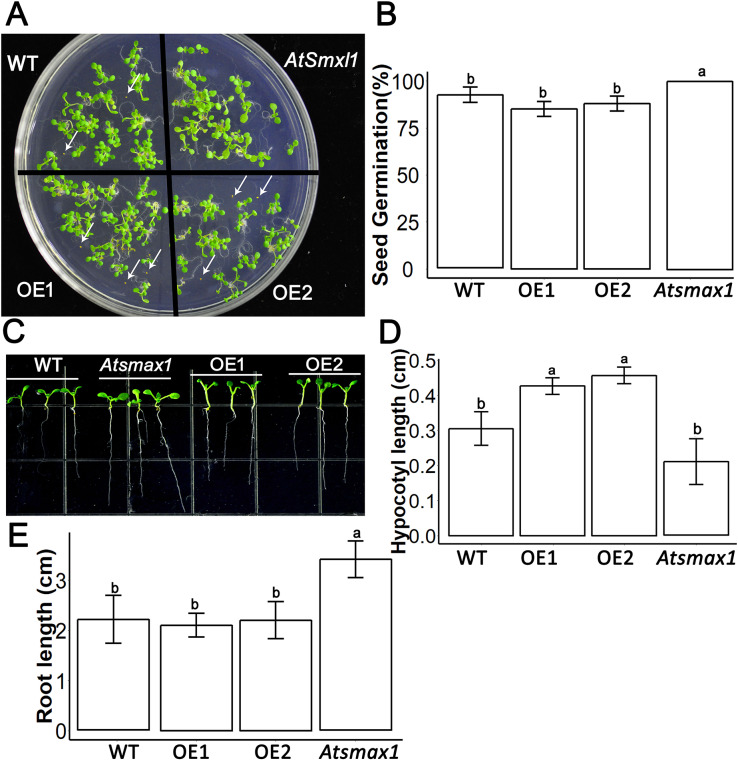
*SsSMAX1* involves seed germination, hypocotyl and root development. (A) Seed germination of *SsSMAX1* transgenic lines; overexpression line 1 (OE1) and 2 (OE2), *Atsmax1*, and wild-type (WT) *Arabidopsis*. Black arrows head represent non-germinated seeds. (B) Graphical presentation of seed germination result of part A, multiple comparisons were made with HSD Tuckey’s test at *p* = 0.5 significant level (*n* = 5). (C) Hypocotyl of wild-type, *Atsmax1*, OE1 and OE2 *Arabidopsis* lines. White arrows are indicated root length difference of *Atsmax1* is greater than the roots of other genotypes. (D) Graphical ANOVA of (C). (E) Root length of WT, *Atsmax1*, OE1 and OE2 after 7 days of germination. All data were analysed with one-way ANOVA, and multiple comparisons were made with HSD Tuckey’s test at *p* = 0.5 significant level (*n* = 15). (A) white bar = 2 cm. (C) white bar = 1 cm.

*SsSMAX1* was found to be overexpressed in the model plant *Arabidopsis thaliana*. Results showed that the ectopic expression of *SsSMAX1* in *Arabidopsis* hindered the seed germination frequency of the transgenic *SsSMAX1* phenotype (Fig. 3A). In contrast, *Atsmax1* function mutants showed quicker germination than wild-type and *SsSMAX1* background seeds (Fig. 3B). Hypocotyl length was increased in *SsSMAX1* background *Arabidopsis* seedlings compared to wild-type and *Atsmax1* seedlings, and *Atsmax1* had the shortest hypocotyl length of the three (Figs. 3C and 3D).

### SsSMAX1 expression in Arabidopsis and its role in plant growth and development

**Figure 4:**
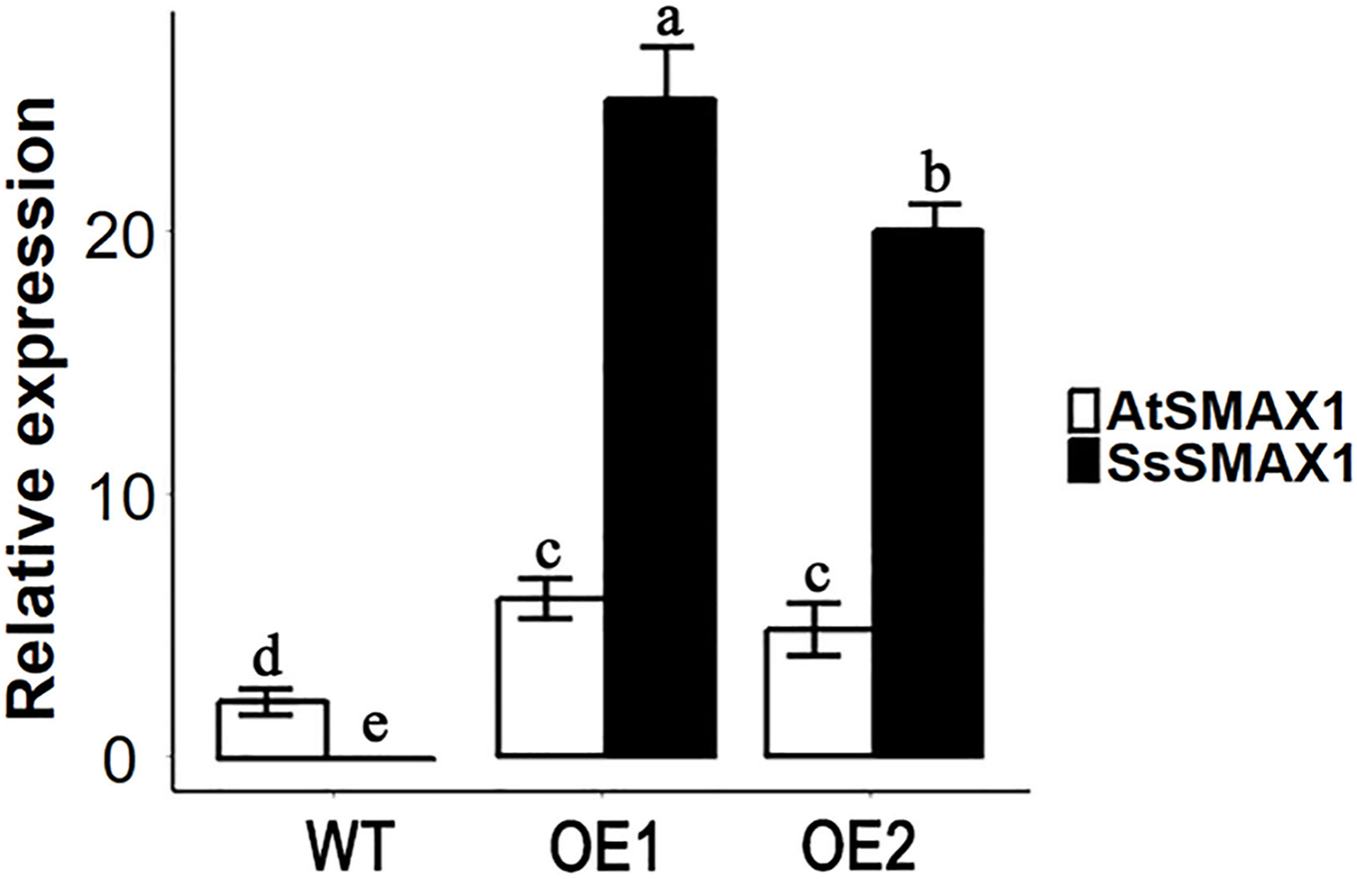
Expression level of *AtSMAX1* and *SsSMAX1* in *Arabidopsis*. The figure shows the expression levels of *AtSMAX1* and *SsSMAX1* in wild-type (WT) and *SsSMAX1* transgenic lines (OE1 and OE2, respectively) of *Arabidopsis*. Leaves of 15-day-old seedlings of wild-type (WT) and *SsSMAX1* transgenic *Arabidopsis* line 1 (OE1) and 2 (OE2) were collected from gene expression analysis. All data were analysed with one-way ANOVA, and multiple comparisons were made with HSD Tuckey’s test at *p* = 0.5 significant level (*n* = 3).

**Figure 5:**
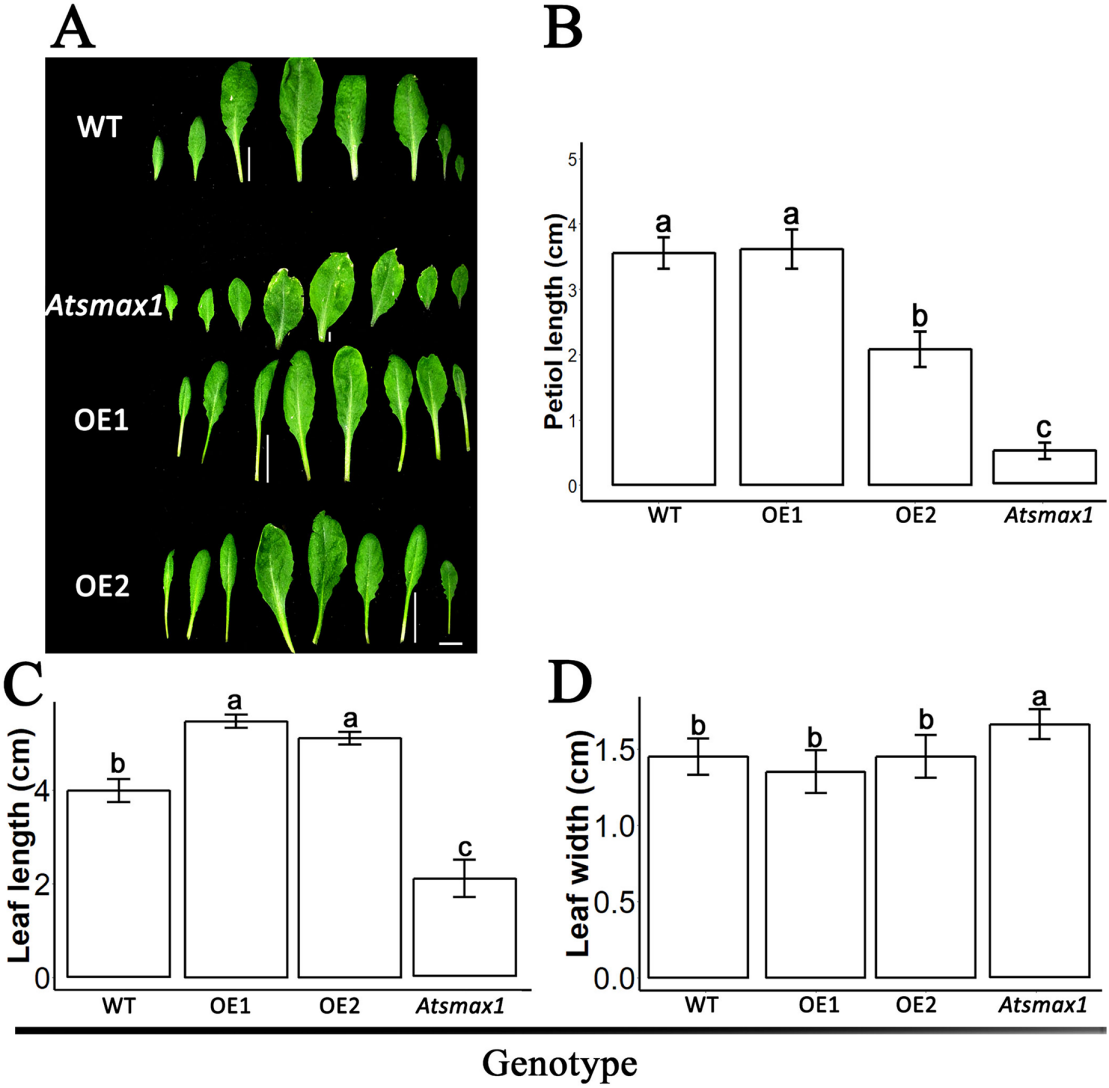
*SsSMAX1* involved in plant leaf development. (A) Leaves of 35-day-old wild-type seedlings (WT), *Atsmax1*, and *SsSMAX1* transgenic *Arabidopsis* lines (OE1 and OE2). The white bar on the basement of the picture = 1 cm. (B–D) Petiole length, leaf length and leaf width, respectively, cm is centimetre. All data were analysed with one-way ANOVA, and multiple comparisons were made with HSD Tuckey’s test at *p* = 0.5 significant level (*n* = 15). (A) white bar =1 cm.

The expression level of *SsSMAX1* was evaluated in *Arabidopsis* by qPCR, and the results showed that *SsSMAX1* was highly expressed in both transgenic lines. Expression levels of *AtSMAX1* were considerably lower than *SsSMAX1* (Fig. 4). A phenotypical analysis showed that *SsSMAX1* and wild-type had more rosette branches than *Atsmax1*. Total number of cauline leaves, inflorescence height, and number of secondary branches were not significantly different between *SsSMAX1*, *Atsmax1*, and wild-type. Leaf width was remarkably higher in *Atsmax1* than in *SsSMAX1* and wild-type (Figs. 5A–5D). Leaf length and petiole length were significantly larger in *SsSMAX1* (Figs. 5A–5D).

## Discussion

*S. sebiferum* has mainly been propagated from seed, but because of deep dormancy, the effectiveness of this strategy has been severely constrained. Previous studies found that KAR_1_ impacts *Sapium sebiferum* seed germination, and that *SMAX1* is widely distributed in seeds and is essential for seed germination and seedling growth in the karrikins pathway. Recent studies suggest that the homologous *SUPPRESSOR OF MAX2 1 (SMAX1)* in *Arabidopsis* and *DWARF53 (D53)* in rice (*Oryza sativa*) are downstream targets of *MAX2* (Jiang et al., 2013; Soundappan et al., 2015; Zhou et al., 2013). *SMAX1* is one of the eight SMXL family proteins involved in KARs and SLs signaling (Soundappan et al., 2015; Stanga et al., 2013). The results of a previous study showed that *SMAX1* is a downstream target of *KAI2*-*MAX2*-mediated signaling of karrikins (Soundappan et al., 2015), but it is still unclear whether *SMAX1* functions are preserved in a perennial woody plant like *S. sebiferum*. This study identified *SMAX1* in *S. sebiferum* and then functionally characterized it by ectopic expression of *SsMAX1* in *Arabidopsis thaliana*.

A bioinformatics analysis revealed that *SsSMAX1* is more similar to *PtSMAX1* than to *AtSMAX1*, and the phylogenetic tree showed that *SsSMAX1* has the same clad as papaya *CpSMAX1*. Both proteins lie subclade to *Nicotiana tabacum SMAX1*, which predicted the conserved functions of *SsSMAX1*. Because *SsSMAX1* is more similar to *PtSMAX1*, they may have similar roles. Previous studies have shown that *Populus trichocarpa* SMAX1 (PtSMAX1) and other proteins are functionally conserved (Sun et al., 2023; Tang et al., 2010). In this study, *SsSMAX1* expression was checked in different parts of the *S. sebiferum* plant, and *SsSMAX1* was phenotypically characterized in *Arabidopsis*. The expression pattern of *SsSMAX1* revealed that it is abundantly expressed in seeds, hypocotyl, and root tips, which is consistent with the findings of previous studies (Nelson et al., 2010; Soundappan et al., 2015; Stanga et al., 2013; Villaécija-Aguilar et al., 2019). These results suggest that *SMAX1* is involved in seed germination, hypocotyl, and root development.

Karrikins was discovered to be involved in *Arabidopsis* seed germination, which requires gibberellic acid synthesis and light. In this study, *SsSMAX1* was ectopically expressed in *Arabidopsis* with the help of a 35S promoter, and seeds of *SsSMAX1* background *Arabidopsis* were slightly more dormant than wild-type *Arabidopsis* seeds. The results of a comparison between the seed germination of *SsSMAX1* phenotypes and the *Atsmax1* functional mutants revealed that *Atsmax1* was non-dormant compared to *SsSMAX1* seeds. These results are consistent with Stanga et al. (2013), who reported that the *Atsmax1* mutant’s seed could germinate early and suppress the dormancy created by the functional mutation of *Atmax2*. A previous study showed that ABA removes the acceleration effect of KARs on germination, and KARs need the biosynthesis of GA to promote seed germination (Nelson et al., 2009). KARs suppress the expression of *IAA1*, which is the IAA response gene; thus, KARs may accelerate seed germination by suppressing the signals of IAA (Nelson et al., 2011). The results of this study showed that ectopic expression of *SsSMAX1* in *Arabidopsis* promoted dormancy in seeds, suggesting that the function of *SsMAX1* is conserved in seed germination.

Karrikin signaling gene *SMAX1* has been reported to regulate *Arabidopsis* hypocotyl elongation by regulating auxin homeostasis (Soundappan et al., 2015). The results of this study showed that the seedlings of *SsSMAX1 OEs* have longer hypocotyl than wild-type *Arabidopsis*. These results support the findings of previous studies that *SMAX1* regulates hypocotyl length and is downstream of *KAI2-MAX2*-mediated signaling of karrikins (Soundappan et al., 2015; Xu, Jinbo & Cai, 2022). These results suggest that the hypocotyl elongation function of *SsSMAX1* is similar to the *SMAX1* gene of other candidate species, such as *Arabidopsis*.

Leaf development, such as leaf width and petiole length, has also been reported as a function of *SMAX1*. The results of this study showed that *SsSMAX1* background *Arabidopsis* has more extended and less broadleaf than wild-type and *Atsmax1* functional mutants. These results are consistent with the results of previous studies, which demonstrated that karrikins *Atsmax1* produced seedlings that had broader leaves than wild-type (Nelson et al., 2012; Soundappan et al., 2015; Stanga et al., 2013). The petiole length of *SsSMAX1* plants was much longer than wild-type and *AtSMAX1* functional mutants. These results are consistent with the results of Stanga et al. (2013) that the enlarged length of the petiole of *Atmax2* was suppressed in *Atsmax1-Atsmax2* double mutant seedlings. A previous study found that the accumulation of *SMAX1* promotes the leaf length/width ratio and petiole length by regulating the expression of the genes involved in auxin transport, the cytokinin signaling pathway, and SL biosynthesis (Zheng et al., 2021). Root growth is necessary to expand the absorptive root surface area and plays a crucial role in plant life. The function of *SsMAX1* has been reported in the development of roots and root hairs. This study showed that ectopic expression of *SsSMAX1* reduced root length and root hairs in *Arabidopsis*, in line with previous studies that showed *SMAX1* regulates root hair elongation by repressing ethylene biosynthesis *via* inhibition of *PIN2* (Carbonnel et al., 2020; Villaécija-Aguilar et al., 2019, 2022). This study’s results suggest that *Sapium sebiferum SMAX1* regulates seedling development, including seed germination, hypocotyl development, root length control, and leaf length and width regulation, thus, *SsSMAX1* is also functionally conserved.

## Conclusion

This is the first study to characterize the *SMAX1* of woody perennial plant, *S. sebiferum*. In alignment with previous studies of different plant species (Carbonnel et al., 2020; Jiang et al., 2013; Soundappan et al., 2015), *SsSMAX1* regulated seed germination, hypocotyl length, petiole length, leaf length and width, and root length in *Arabidopsis*, revealing that *SMAX1* has conserved functions in *S. sebiferum*. This study also provides the foundation for an understanding of the role of *SMAX1* in other plant specie*s*.

## Supplemental Information

10.7717/peerj.16610/supp-1 Supplemental Information 1 *SsSMAX1* gene’s full-length CDS and protein sequences. *SsSMAX1* gene’s full-length CDS and protein sequences

10.7717/peerj.16610/supp-2 Supplemental Information 2 List of Primers. Primers used for qPCR were designed by using primer Premier 6. The T_m_ of the primers was between 59.0 and 61.0° C . Primers used for qPCR were designed by using primer Premier 5.

10.7717/peerj.16610/supp-3 Supplemental Information 3 Supplementary Figure 1: Diagram of over-expression vector. *SsSMAX1* was cloned by using primers given in supplementary table 1. Ligated to blunt end vector, sequenced, and then digested at Sal1 and Sma1 positions. Overexpression vector pOCA30 was also double-digested at Sal1 and Sma1 positions. The Full-length *SsSMAX1* gene was ligated to pOCA30.

10.7717/peerj.16610/supp-4 Supplemental Information 4 Figure 2 data. Gene expression determination by qPCR. Data represent the 2^−∆∆Ct^ value of each gene. *SsACTIN2* was used as a reference gene.

10.7717/peerj.16610/supp-5 Supplemental Information 5 Figure 3 data. Hypocotyl length, Root length, Seed germination of *SsSMAX1* transgenic lines; overexpression 1 (OE1), 2 (OE2), *Atsmxl1*, and wild-type (WT)

10.7717/peerj.16610/supp-6 Supplemental Information 6 Figure 4 data. Expression levels in wild-type and *SsSMAX1* transgenic line 1 and line 2 (OE1 and OE2 respectively). Leaves of 15-day-old seedlings of wild-type (WT), *AtSMAX1*, and *SsSMAX1* transgenic *Arabidopsis* line 1(OE1) and 2 (OE2) were collected from gene expression analysis.

10.7717/peerj.16610/supp-7 Supplemental Information 7 Figure 5 data. Leaf length, Leaf width, and Petiol length of *SsSMAX1* transgenic lines; overexpression 1 (OE1), 2 (OE2), *Atsmxl1*, and wild-type (WT)

## Additional Information and Declarations

### DNA Deposition

The following information was supplied regarding the deposition of DNA sequences: The sequences are available at GenBank: SRX656554.

### Data Availability

The following information was supplied regarding data availability: The raw measurements are available in the Supplemental Files.
