# Supplementary material for: Identification and characterization of the karrikins signaling gene SsSMAX1 in Sapium sebiferum
Source: PeerJ. 2023 Dec 8;11:e16610. doi: 10.7717/peerj.16610 (PMC10712317; doi:10.7717/peerj.16610)
Supplement: Supplemental Information 1 — SsSMAX1 gene’s full-length CDS and protein sequences [file peerj-11-16610-s001.docx]

**CDS and Protein sequence of *SsSMAX1***

atgagagctggacttagtacgattcagcaaacgctgaccccagaggcggcgagtgtttta

M R A G L S T I Q Q T L T P E A A S V L

aaccactcaattgcggaagcgactcgccggaatcatggtcagactacgccgcttcatgtt

N H S I A E A T R R N H G Q T T P L H V

gcggcgacgctcttggcttctccttctgggtatcttcgacaagcttgtattaaatcgcac

A A T L L A S P S G Y L R Q A C I K S H

ccaaattcttctcatcctcttcagtgcagagcccttgagctctgctttagcgtggctcta

P N S S H P L Q C R A L E L C F S V A L

gaacggctacccactgcgcaaaatcttagcccaggtcatgacccaccaatctccaatgca

E R L P T A Q N L S P G H D P P I S N A

ttaatggcggcactgaagcgtgctcaggctcaccagcggcggggtttccctgaacagcag

L M A A L K R A Q A H Q R R G F P E Q Q

caacagcctttgctcgccgtcaaagttgagctcgagcagctaattatatcaattttagat

Q Q P L L A V K V E L E Q L I I S I L D

gatccaagtgtgagcagagttatgagagaagctagtttttcaagcccagctgttaaggcc

D P S V S R V M R E A S F S S P A V K A

acaattgagcagtctcttaactcttcatcaaattctgcagcctcagcctcaaattcaagc

T I E Q S L N S S S N S A A S A S N S S

tcatttggattcgggtttcgatctcctgcagcagtaccagtgcctacaccaacaacaaat

S F G F G F R S P A A V P V P T P T T N

cggaatttatatgtgaatcccagattgcagcaagggaatatagcccaatctgggcagcat

R N L Y V N P R L Q Q G N I A Q S G Q H

aggaatgaggaagttaagcgagtggtggatattttgttgaagaacaggaagaggaaccct

R N E E V K R V V D I L L K N R K R N P

gttctggtcggagagttggagccggagatggtggtaaaggaggtcttgaaaagaatagag

V L V G E L E P E M V V K E V L K R I E

aataaagaattaggagatgggttgttaaagaatgtgcaagtgattcatttggaaaaggat

N K E L G D G L L K N V Q V I H L E K D

ttttttgataaagctcaaatacccacaaagattatagaattagggggtttaatagggaat

F F D K A Q I P T K I I E L G G L I G N

ttagattgcgaaggagtaattctcgatttgggtgatttaaaatggctagtggaacagccg

L D C E G V I L D L G D L K W L V E Q P

gtgagctttactggtactggtggcggtgtgcagcagcaacagcagattatttcagaggct

V S F T G T G G G V Q Q Q Q Q I I S E A

ggccgggctgcggtgacagagatggggaaactgttagcaagatttggagagaaaagggtt

G R A A V T E M G K L L A R F G E K R V

tggttgataggtactgctacttgtgaaacatatttaaggtgccaagtttatcatccttca

W L I G T A T C E T Y L R C Q V Y H P S

atggagaatgattgggatctgcaggctgttccaattgctccaagaactcttgcaggaaga

M E N D W D L Q A V P I A P R T L A G R

tttcctaggattggaaccaatgggattcttaacagctcggtcgagtctttctcgcctctg

F P R I G T N G I L N S S V E S F S P L

aagggctttccaactgtcacacctaatctaccaaagtcaggtcctgctcgaaacacaagt

K G F P T V T P N L P K S G P A R N T S

tgttgcccacagtgtatgcagaattatgagcaagagcttgccagaattgaatcaaaagat

C C P Q C M Q N Y E Q E L A R I E S K D

tccgataactcttcttctgaatttaaatcagaagcaacccattcgcagctgccgcagtgg

S D N S S S E F K S E A T H S Q L P Q W

ttgaaaaatgccaagtctcaagatggtgatgataaaacatcggataataaggaacacaag

L K N A K S Q D G D D K T S D N K E H K

agtcaagagttgcagaagaaatggcatgatgcgtgtaagcaacttcatcctggctatcat

S Q E L Q K K W H D A C K Q L H P G Y H

cagcccaatgttagccctgagagactcacacaaacggcgctctctacatcaagtttgtat

Q P N V S P E R L T Q T A L S T S S L Y

aatccaaacctgctttcttgccaacctttgatgccgaagataggtttgaataaaagtctt

N P N L L S C Q P L M P K I G L N K S L

gctgcaactacactgttgaacccaaatttggtgcccagccaatcacctgctcgcactatt

A A T T L L N P N L V P S Q S P A R T I

actccaccaggaagccctgtgagaacagatttggttcttgggcggccaaagagcaaagac

T P P G S P V R T D L V L G R P K S K D

aacactgctgagaaagtccacgaagagcgaactaaaaatttattgggttgtgttgcttct

N T A E K V H E E R T K N L L G C V A S

gagccacaaataaagtttactgaattgcaggtcaacaagctagatgctgactcgttcaag

E P Q I K F T E L Q V N K L D A D S F K

aggctccttaagggtcttattgacaaggtgtggtggcagcgagaagcagcatccgctgtg

R L L K G L I D K V W W Q R E A A S A V

gctacaactgtgacacaatgcaaaatgggtaatggtaaacggcggggaggtggatcaaag

A T T V T Q C K M G N G K R R G G G S K

ggtgacatttggctattgttcacaggtccagacagggatggcaagaagaggatggcatca

G D I W L L F T G P D R D G K K R M A S

gctctttcagaccttgtatgcggggcctgtccaataatggtttctcttggttcccgtcgt

A L S D L V C G A C P I M V S L G S R R

gacgatggggaacctactatgaatttccgtggtaaaacagcgctagatcgtatagcggag

D D G E P T M N F R G K T A L D R I A E

gctgttagaaggaacccattttcagtagtcatgctggaggatatcgatgaagcagatgta

A V R R N P F S V V M L E D I D E A D V

ctagttacagggagcataaagcgagccatggaaagaggtcggctttctgactcccatggc

L V T G S I K R A M E R G R L S D S H G

cgtgaaatcagtctcgggaatgtcattttcatccttactgcaaattgggtaccagacaat

R E I S L G N V I F I L T A N W V P D N

ctgaaattcctaatgagtggtgtttcacttgatgagaacaagctcgcaaatttggcagga

L K F L M S G V S L D E N K L A N L A G

ggagggtggcaattgaaactatccctctatgagaaaacagcaaagcgacgagccaattgg

G G W Q L K L S L Y E K T A K R R A N W

ctgcatgacgaagagaggcctgcaaagccaaggaaagactcaggatcagcactatcattt

L H D E E R P A K P R K D S G S A L S F

gacctgaacgaagcagctgatgctgaggaggataaagcggatggctctcgcaattcaagt

D L N E A A D A E E D K A D G S R N S S

gatcttacaattgatcatgaagatgagaatgttctcaataacagtctactaatgccaaca

D L T I D H E D E N V L N N S L L M P T

acttcatcagtacctcgagagctgctcaatgcagtcgatgatgatatagttttcaaacct

T S S V P R E L L N A V D D D I V F K P

atagattttagctctattcgatccgaggtttccaactctataaccaaaaggttctgcacc

I D F S S I R S E V S N S I T K R F C T

attatgagcgaggaggggatccagttggaaattaaagaggaggcacttgaaaaggttgcc

I M S E E G I Q L E I K E E A L E K V A

ggagggttatggttaagccgaacgagtttagaagaattcactgagaaagtagtggttcca

G G L W L S R T S L E E F T E K V V V P

agcattcgccagctcaaattgcagttgccaacatctgcagatgagtccatggttgtgagg

S I R Q L K L Q L P T S A D E S M V V R

ctcgaattggacagcgattcagctcaacggagccatggagattggctgccgaacagtgtg

L E L D S D S A Q R S H G D W L P N S V

agagtggtggtcgatgggttgtga

R V V V D G L -
