# Supplementary material for: Identification and characterization of the karrikins signaling gene SsSMAX1 in Sapium sebiferum
Source: PeerJ. 2023 Dec 8;11:e16610. doi: 10.7717/peerj.16610 (PMC10712317; doi:10.7717/peerj.16610)
Supplement: Supplemental Information 2 — Primers used for qPCR were designed by using primer Premier 6. The Tm of the primers was between 59.0 and 61.0° C . Primers used for qPCR were designed by using primer Premier 5. [file peerj-11-16610-s002.docx]

| *AtSMAX1* | Sense Primer | GAGCAACCGTACAGTCACCGTAAG |
| --- | --- | --- |
|  | Anti-sense Primer | CGGTAGCAGCAGCGATTACAGAAT |
| *AtSMAX2* | Sense Primer | TCATCCATCCTAGAACTAGAACCAAGACT |
|  | Anti-sense Primer | TGGTGGTGGTAGTGGAACTTGTTATTC |
| *SsACTIN2* | Sense Primer | GTATCGTGTTGGATTCTGGTGATGGT |
|  | Anti-sense Primer | CGGCAGTGGTGGTGAAGGAGTA |
| *AtACTIN2* | Sense Primer | TCATCCATCCTAGAACTAGAACCAAGACT |
|  | Anti-sense Primer | TGGTGGTGGTAGTGGAACTTGTTATTC |
| *SsSMAX1 full length* | Sense Primer | TCATCCATCCTAGAACTAGAACCAAGACT |
|  | Anti-sense Primer | TCATCCATCCTAGAACTAGAACCAAGACT |
| *SsSMAX1 qPCR* | Sense Primer | TCATCCATCCTAGAACTAGAACCAAGACT |
|  | Anti-sense Primer | TGGTGGTGGTAGTGGAACTTGTTATTC |

**Supplementary Table 1**

**List of primers used for qPCR, and gene cloning**
