# Supplementary figures and images for: Identification and characterization of the karrikins signaling gene SsSMAX1 in Sapium sebiferum
Source: PeerJ. 2023 Dec 8;11:e16610. doi: 10.7717/peerj.16610 (PMC10712317; doi:10.7717/peerj.16610)

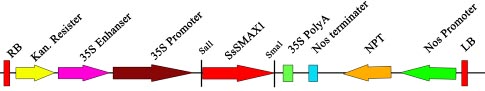

Supplement: Supplemental Information 3 — SsSMAX1 was cloned by using primers given in supplementary table 1. Ligated to blunt end vector, sequenced, and then digested at Sal1 and Sma1 positions. Overexpression vector pOCA30 was also double-digested at Sal1 and Sma1 positions. The Full-length SsSMAX1 gene was ligated to pOCA30. [file peerj-11-16610-s003.jpg]
